# Formation of starch in plant cells

**DOI:** 10.1007/s00018-016-2250-x

**Published:** 2016-05-11

**Authors:** Barbara Pfister, Samuel C. Zeeman

**Affiliations:** Department of Biology, ETH Zurich, 8092 Zurich, Switzerland

**Keywords:** *Arabidopsis thaliana*, Amylopectin, Amylose, Protein phosphorylation, Protein complex formation

## Abstract

Starch-rich crops form the basis of our nutrition, but plants have still to yield all their secrets as to how they make this vital substance. Great progress has been made by studying both crop and model systems, and we approach the point of knowing the enzymatic machinery responsible for creating the massive, insoluble starch granules found in plant tissues. Here, we summarize our current understanding of these biosynthetic enzymes, highlighting recent progress in elucidating their specific functions. Yet, in many ways we have only scratched the surface: much uncertainty remains about how these components function together and are controlled. We flag-up recent observations suggesting a significant degree of flexibility during the synthesis of starch and that previously unsuspected non-enzymatic proteins may have a role. We conclude that starch research is not yet a mature subject and that novel experimental and theoretical approaches will be important to advance the field.

## Introduction

Starch is an insoluble, non-structural carbohydrate composed of α-glucose polymers. It is synthesized by plants and algae to store energy in a dense, osmotically inert form. Starch has significant value for humans: it serves as the main carbohydrate source in an equilibrated diet and as a renewable raw material for industry. For instance, starch is extensively used as a thickener and texturizer in processed foods, as it gelatinizes to form pastes when heated in water. Starch pastes also have innumerable uses in the non-food sector, such as in the production of paper and board [1, 2], of biodegradable plastics and packaging materials [3] amongst others.

Based on its biological functions, starch is often categorized into two types: transitory starch and storage starch. The starch which is synthesized in the leaves directly from photosynthates during the day is typically defined as transitory starch, since it is degraded in the following night to sustain metabolism, energy production and biosynthesis in the absence of photosynthesis. If this night-time carbohydrate supply is reduced—for instance in mutants impaired in starch synthesis—plants grow more slowly and experience acute starvation [4]. The starch in non-photosynthetic tissues, such as seeds, stems, roots or tubers, is generally stored for longer periods and regarded as storage starch. Remobilization takes place during germination, sprouting or regrowth, again when photosynthesis cannot meet the demand for energy and carbon skeletons for biosynthesis. Also mutants with perturbation in storage starch biosynthesis are often disadvantaged, and mutant seeds with low or no starch may even be inviable [5, 6]. It is this storage starch that we consume as our food and extract for industrial uses—it can account for 70–80 % of the dry weight in wheat grains and cassava roots [7, 8].

Starches from different botanical sources vary in terms of their functional properties (e.g., gelatinization onset temperature, final viscosity of paste, formation of two-phase pastes or paste stickiness) and thus in their end-uses. This variation stems from differences in the structure of starch, such as the size of starch granules, their composition, and molecular architecture of the constituent polymers [9]. Still, extracted starch often needs to be modified using costly and sometimes waste-generating chemical, physical or enzymatic treatments to confer or enhance the required functional properties [10]. Starch structure also influences its digestibility in the gut. Those with reduced digestibility (resistant starch), such as high-amylose starches, are increasingly valued due to their health-promoting effects, potentially serving as a preventive measure against conditions such as colorectal cancer and diabetes [11]. Understanding starch biosynthesis and its relationships to structure and functionality is of enormous interest as it represents a prerequisite for the targeted improvement of starch crops.

This review focuses on the mechanisms of starch biosynthesis and seeks to provide a broad overview of our current knowledge, while highlighting recent advances. Significant steps in our basic knowledge have been made through the analyses of model systems such as the plant *Arabidopsis thaliana* and the single-celled green alga *Chlamydomonas reinhardtii.* Although their starches have no direct industrial value, many aspects of starch biosynthesis appear to be widely conserved within the Viridiplantae clade. Thus, discoveries made in these systems are likely to have broad relevance. It is nevertheless always important to bear in mind the cellular and metabolic context in which starch is made. Variation in conditions between tissues and species can have a strong influence on the amount and structure of starch. Such differences may explain why, in some cases, different phenotypes result from similar genetic perturbations. In the long run it will be important to understand both the basic starch-biosynthetic process and tissue-specific factors that affect it.

## The structure of starch

**Fig. 1:**
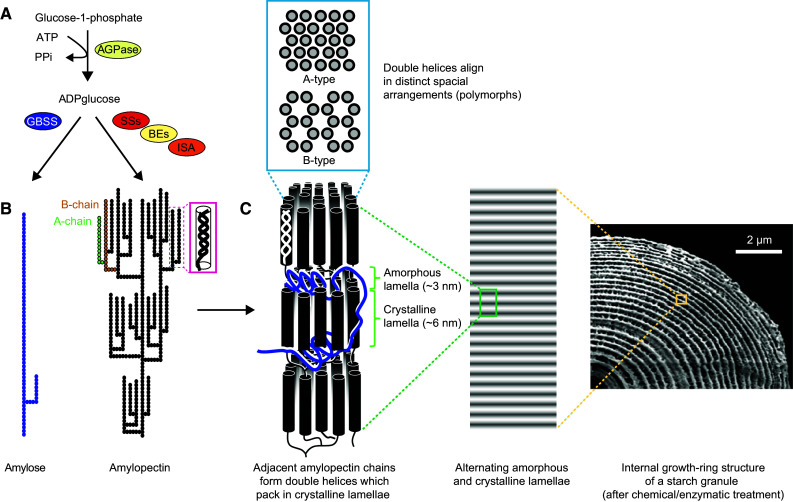
The structure and biosynthesis of starch. **a** Overview of the core starch biosynthesis pathway. ADPglucose pyrophosphorylase (AGPase) produces ADPglucose, the substrate of starch synthases (SSs). Granule-bound starch synthase (GBSS) synthesizes amylose, while soluble SSs, branching enzymes (BEs) and isoamylase-type debranching enzyme (ISA) collectively synthesize amylopectin. **b** Molecular structure of amylose and amylopectin (according to the cluster model), showing its branching pattern and formation of secondary structures. *Filled, joined circles* represent individual glucosyl residues. **c** High-order alignment of amylopectin double helices. Each growth ring (*right*) has a thickness of ca. 200–400 nm and contains a semi-crystalline region and an amorphous region. The semi-crystalline region consists of alternating crystalline lamellae (containing the linear parts of the chains) and amorphous lamellae (containing most of the branch points) which stack with a periodicity of ~9–10.5 nm (*middle*). Depending on the exact architecture of the amylopectin giving rise to the clusters, the double helices either arrange as densely packed A-type polymorph or less dense hexagonal B-type polymorph (*top*). A mixture of A and B is also possible and named C-type polymorph (not shown). Figure composed using parts from [30] (with permission from Elsevier) and [284] (thearabidopsisbook.org; Copyright American Society of Plant Biologists)

Starch consists of the two glucose polymers amylopectin and amylose, which together form insoluble, semi-crystalline starch granules (Fig. 1; see [12] for a comprehensive review). Both polymers are made of α-1,4-linked glucan chains connected with α-1,6-branch points, but their structure and biosynthesis are distinct. Amylopectin accounts for 75–90 % of wild-type starches, has a degree of polymerization (DP) of ~10^5^ and a branching level of 4–5 % (i.e., 4–5 % of its linkages are α-1,6-branch points) [13]. Amylopectin makes up the structural framework and underlies the semi-crystalline nature of starch. Amylose is considerably smaller and only lightly branched [13]. It is believed to fill spaces in the semi-crystalline matrix formed by amylopectin, probably rendering the starch granule denser.

The relatively simple chemical nature of starch is in stark contrast to the structural complexity of the final starch granule. This chemical simplicity makes it difficult to obtain definitive structural information about the glucans. Instead, the techniques used to determine the abundance of structural units (e.g., numbers of branch points or chain lengths) tend to deliver average measures that mask structural heterogeneity. Such limitations mean that we rely on structural models of starches. Inevitably this can impact on the interpretation of data, such as substrate preferences of enzymes or mutant phenotypes, and affects our ability to unambiguously ascribe specific enzyme functions. Still, for many enzymes, data of different types have helped to build a consensus view of their role in starch synthesis.

### Molecular structure of amylopectin

It is generally accepted the branching frequency and pattern in amylopectin is non-random. Within each molecule, the constituent chains are categorized according to their connection to other chains: A-chains are the external chains that carry no branches themselves while B-chains are those that carry one or more branches. The C-chain is the single B-chain chain per molecule having a free reducing end. In the models of amylopectin, the branch points are concentrated in certain regions from which the linear chains segments extend to form clusters (Fig. 1). The frequency distribution of chain lengths (chain-length distributions or CLDs), deduced from the analysis of debranched starches, shows that most chains are of between 10 and 20 glucose units. These are considered to be the A- and B_1_-chains (B-chains that participate in the formation of one cluster). However, there are also longer chains, which are thought to form connections between different clusters. These are generally believed to be oriented in the same orientation as the cluster-filling A- and B_1_-chains (cluster model [14, 15]) and assigned as B_2_-, B_3_-, B_4_-chains for chains spanning two, three or four clusters, respectively; [16]. They could, however, also be oriented perpendicularly to the clusters (backbone model [17]).

X-ray scattering and electron microscopic analyses suggest that clusters stack with a periodicity of ~9–10 nm [18, 19]. X-ray diffraction patterns further reveal that the neighboring linear chain segments within clusters form parallel double helices, with each complete turn having 6 glucose units per chain and a period of 2.1 nm. The double helices align in the dense A-type polymorph or the less dense (and more hydrated) B-type polymorph [20, 21] (Fig. 1c). Starches containing mixtures of A- and B-type polymorphs are also observed and named C-type polymorph. A-type polymorphs are typical of cereal grains and B-type polymorphs of tuber starches. The factors responsible for determining the polymorph type are not fully understood, however.

### High-order structures of starch

Various microscopic analyses suggest levels of organization beyond the 9-nm-repeat (Fig. 1c). Some of the earliest drawings and light micrographs of starch granules showed concentric layers within the granules. These were called ‘growth rings’ due to the superficial similarity in appearance to the growth rings of trees. Treating cracked starch granules with α-amylase or acid, which removes the less crystalline regions, and analysis with scanning electron microscopy clearly reveal growth rings as a repeating layered structure with a period of a few hundred nanometers. Each of these resistant layers is thought to be composed of numerous 9-nm-repeats. The susceptible amorphous region is presumed to have a lower degree of order [22]. In addition to the growth ring structure, spherical blocklets with a diameter between 20 and 500 nm have been observed in the semi-crystalline regions of starches [23]. These might represent a left-handed amylopectin super-helix, which was proposed by Oostergetel and van Bruggen [24] based on electron optical tomography and cryo electron diffraction analyses. While some of the structural features of starch are widely accepted, such as the formation and packing of double helices and the presence of growth rings, others remain less well understood. The potential introduction of artefacts during sample preparation for many of the techniques applied needs to borne in mind.

Starch granules from different species and tissues vary greatly in size and shape, ranging from relatively small particles of 0.5–2 µm in diameter in amaranth seeds and flat disks in Arabidopsis leaves to smooth spheres of up to 100 µm in tuberous roots [25, 26]. Granules contain small amounts of protein (typically 0.1–0.7 %), which is mostly the granule-bound starch synthase (GBSS) that makes amylose, but also other amylopectin synthesizing enzymes, such as other starch synthases (SSs) and starch-branching enzymes (BEs) [27, 28]. Many starches further contain traces of lipids and phosphate groups (covalently linked at the C6 or C3 position of glucose) [27]. The phosphorylation level of cereal starches is extremely low. In Arabidopsis leaf starch it is around 0.05 % (i.e., around one per 2000 glucose units is phosphorylated), while in tuber starches it can be many times higher (~0.5 % in potato). Phosphorylation appears to be confined to amylopectin and enriched in the amorphous regions [29]. A high phosphate content is an industrially relevant trait as it is associated with an increased granule hydration and lowered crystallinity, yielding starch pastes with higher transparency, viscosity and freeze–thaw stability ([30] and references therein).

## The enzymes of starch biosynthesis

Starch is synthesized in the plastids—chloroplasts in leaves or specialized amyloplasts in the starch-storing tissues of staple crops. In red algae and glaucophytes, the situation is different; their so-called floridean starch is synthesized in the cytosol via a pathway which appears to be mechanistically different from that in plants and green algae [31, 32]. Starch synthesis in plants involves three major enzyme activities: First, SSs elongate the non-reducing ends of glucose chains using adenosine 5′-diphosphate-glucose (ADPglucose) as glucosyl donor; second, BEs create branches from existing chains via glucanotransferase reactions; and third, debranching enzymes (DBEs) hydrolyze some of the branches again (Fig. 1a). Although presented in a sequential manner, it is important to perceive it as a simultaneous, interdependent process. The starch-biosynthetic enzymes are well conserved between different plant species, suggesting a common origin [33]. The basic mechanism of starch biosynthesis resembles that of glycogen, the water-soluble α-1,4 and α-1,6-linked glucose polymer synthesized in many bacteria, fungi and animals. However, as described below, there is more complexity in starch biosynthesis in terms of duplication and specialization of SSs and BEs, the recruitment of additional enzymes (i.e., DBEs) and other recently described proteins that may contribute to the formation of the semi-crystalline starch granule.

### ADPglucose pyrophosphorylase (AGPase) provides the substrate for starch biosynthesis

Starch synthesis starts with the production of ADPglucose, the substrate for SSs. In photosynthetically active chloroplasts of leaves, the generation of ADPglucose is directly linked to the Calvin–Benson cycle through conversion of fructose-6-phosphate to glucose-6-phosphate (Glc-6-P) (catalyzed by phosphoglucose isomerase) through to glucose-1-phosphate (Glc-1-P) (catalyzed by phosphoglucomutase). AGPase (EC 2.7.7.27) then catalyzes the conversion of Glc-1-P and ATP to ADPglucose and pyrophosphate (PP_i_). Via this pathway approximately 30–50 % of photoassimilates of Arabidopsis leaves are partitioned into starch [34]. Each of the aforementioned reactions is thermodynamically reversible. However, in vivo, the PP_i_ product of the last reaction is further metabolized by plastidial alkaline pyrophosphatase, hydrolyzing it to yield two molecules of orthophosphate (P_i_) [35, 36]. This renders the synthesis of ADPglucose in the chloroplast essentially irreversible. Indeed, Arabidopsis mutants deficient in SSIV (described below), which are unable to utilize ADPglucose for starch synthesis, display a strong impact on photosynthetic metabolism, attributed to the accumulation of ADPglucose and the consequential depletion of the adenylate pool [37].

The synthesis of ADPglucose is similar in heterotrophic tissues where sucrose is imported from source tissues and metabolized to produce hexose phosphates in the cytosol. For starch biosynthesis, both hexose phosphates (typically Glc-6-P, although Glc-1-P transport has also been reported) and ATP are transported into the amyloplast to serve as substrate for the synthesis of ADPglucose [38, 39]. Hexose phosphate transport occurs in exchange for P_i_, while ATP transport occurs in exchange for ADP and P_i_. In the cereal endosperm, the pathway differs: here the major AGPase activity is found in the cytosol and ADPglucose is imported directly into the plastid via a dedicated, cereal-specific subclass of adenine nucleotide transporter [40–43].

The synthesis of ADPglucose by AGPase is often regarded as the “committed step” of starch synthesis. There is appreciable evidence that the step is regulated both at the transcriptional and post-translational levels, which has been reviewed in detail elsewhere [44]. Briefly, AGPase is a heterotetramer consisting of two large regulatory subunits and two small catalytic subunits. In many cases, the enzyme has been demonstrated to be allosterically activated by 3-phosphoglycerate and inhibited by P_i_ (e.g. [45, 46]). The enzyme is furthermore sensitive to redox regulation via the reduction of a intermolecular disulfide bridge that forms between cysteine residues of the small subunit [47–49]. Together, these regulatory features are thought to ensure that ADPglucose, and thus starch, is only made when there are sufficient substrates. Many attempts have been made to promote the flux towards starch by expressing unregulated AGPase from *Escherichia coli* or *planta* (e.g., [50–57]). This has resulted in increased starch content in at least one potato variety [50], increased overall grain yield in maize [52, 56] and wheat [53] and increased tuberous root biomass in cassava [55] (reviewed in [58]).

Although the above-mentioned pathway of ADPglucose production is well accepted, other mechanisms for the production of ADPglucose have been proposed (see [34] and references therein). These alternate pathways, however, require validation.

### The domain structure of starch synthases (SSs)

SSs (ADPglucose:1,4-α-d-glucan 4-α-d-glucosyltransferases; EC 2.4.1.21) belong to the glycosyltransferase (GT) family 5 (CAZy [59]). They catalyze the transfer of the glucosyl moiety of ADPglucose to the non-reducing end (here the C4 position) of an existing glucosyl chain, creating an α-1,4 bond and elongating the chain. Five SS classes are involved in starch biosynthesis: four are soluble in the stroma (or partially bound to the granule) and one is almost exclusively granule bound. The soluble SSs (SSI, SSII, SSIII and SSIV) are involved in amylopectin synthesis while the granule-bound SS (GBSS), is responsible for amylose synthesis. There is an additional putative SS class named SSV that is related in sequence to SSIV but has not yet been functionally characterized [60].

**Fig. 2:**
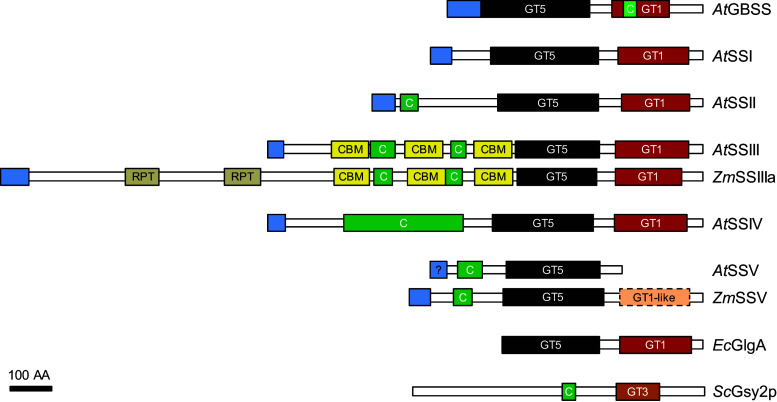
The domain structure of starch synthase (SS) classes. SSs from Arabidopsis (*At*) compared with glycogen synthases from *E. coli* (*Ec*) and budding yeast (*Sc*). Maize (*Zm*) SSIIIa and SSV are included as they differ in their structures compared with the Arabidopsis orthologs. Shown are plastidial transit peptides (N-terminal *blue boxes*), internal repeats (*gray boxes*, RPT), carbohydrate-binding modules of family 25 (*yellow boxes*, CBM), coiled-coil domains (*green boxes*, C), glycosyltransferase-5 domains (*black boxes*, GT5), glycosyltransferase-1 domains (*red boxes*, GT1) and a glycosyltransferase-3 domain (*orange box*, GT3). Transit peptides were predicted with ChloroP [285], coiled-coil motifs with Paircoil2 ([286]; *p* value < 0.05, 21 amino acids minimal length) and all other motifs with SMART. Note that the domain length and annotation depend on the database queried. For example, the GT3 domain of *Sc*Gsy2P is identified as a GT1 domain by SMART and was manually re-assigned as GT3 here [287]. Whereas *Sc*Gsy2p is a GT3 family glycosyltransferase and uses UDPglucose as substrate, all other shown synthases are GT5 family glycosyltransferases and use ADPglucose as substrate. The N-terminal regions of SSIII containing the coiled-coil motifs and CBMs are highly conserved among various orthologs, but further contain internal repeats in some cases (e.g., in barley and wheat SSIIIa). Orthologs of SSII often showed weaker or no coiled-coil predictions. *At*SSV has a weakly predicted chloroplast transit peptide, but lacks the GT1 domain and is of unknown function. SSV from most other species, however, have a C-terminal extension including a stretch that was designated as a putative GT1-like domain [60]. *Bar* 100 amino acids (AA)

SSs consist of a highly conserved C-terminal catalytic domain and a variable N-terminal extension (Fig. 2). The catalytic domain is conserved between SSs and bacterial glycogen synthases, which also use ADPglucose as substrate, and contains both a GT5 and a GT1 domain (CAZy; [61]). According to the crystal structures of *Agrobacterium tumefaciens* and *E. coli* glycogen synthases, the rice GBSSI and barley SSI, the catalytic domain adopts a GT-B fold, with the active site in a cleft between the two GT domains [62–65]. Binding of ADPglucose probably involves one or more conserved Lys-X-Gly-Gly motifs [66–68] and other conserved charged/polar residues [62, 69–72]. The N-terminal extensions of SS classes are dissimilar. In the case of SSIII and SSIV, these extensions were shown to be involved in protein–protein interactions, potentially via conserved coiled-coil motifs [73–75]. The N-terminal part of SSIII also contains three conserved carbohydrate-binding modules (CBMs) that are involved in substrate binding [76, 77].

Gene duplications have resulted in multiple isoforms of some enzymes. The encoded proteins have a high degree of sequence similarity but are often differentially expressed, with specific isoforms predominating in the endosperm or vegetative tissues [78–84]. For example, several isoforms exist for each SS class in cereals (except for SSI and SSV). The “a” isoforms of SSII and SSIII appear to be the predominant isoforms in the endosperm, based on expression and mutant studies (hereafter, we refer to these “a” isoforms of SSII and SSIII unless otherwise stated). In other species, including Arabidopsis and plants having storage starch-filled organs (e.g. potato), there is only one isoform for each class.

### Granule-bound starch synthase (GBSS) synthesizes amylose

Mutants with reduced or no GBSS activity, so-called *waxy* lines, typically produce less or no amylose, respectively, for instance in the endosperms of maize [85, 86], rice [87, 88], wheat [89], barley [90] and amaranth [91], cassava roots [8], potato [92, 93], pea seeds [94], Arabidopsis leaves [95] and *C. reinhardtii* [96, 97]. This suggests that GBSS is responsible for the synthesis of amylose and that no other synthase can replace it in this function.

It is likely that GBSS synthesizes amylose within the granular matrix formed by amylopectin. Monitoring the distribution of amylose over time in potato lines in which GBSS expression and amylose contents were repressed to low levels, suggested that amylose was more apparent toward the center of the starch granule and that this amylose-containing core grows together with the granule [98]. It is important to realize that although insoluble, the granule is hydrated and small molecules such as ADPglucose can apparently diffuse into the matrix and be used by granule-bound proteins [99]. There is in vitro evidence that GBSS acts in a processive rather than distributive manner, preferentially adding glucose units to the same chain instead of switching between chains [68, 99]. GBSS activity also strongly increased when assayed in amylopectin concentrations high enough for spontaneous glucan crystallization to occur [68]. Thus, GBSS may synthesize amylose by elongating individual glucan chains in the environment surrounding crystalline or crystallizing amylopectin. Its product probably is well protected from branching activity, explaining why it is largely linear. The nature of the primer used for amylose synthesis is not fully resolved. Radio-labeling of *C. reinhardtii* starch granules suggested that GBSS first elongates amylopectin chains and then releases these chains to the amylose fraction [100]. This may be different in vascular plants, however, as no transfer of radioactive label from amylopectin to amylose was observed in Arabidopsis [101]. Another primer could be malto-oligosaccharides, the presence of which was shown to increase GBSS activity and increase its specificity toward amylose synthesis (as opposed to the elongation of amylopectin chains) both in vitro [102] and in vivo [101].

Although being almost completely granule bound [103], GBSS does not contain any predicted starch-binding domains (Fig. 2). Recently, a conserved starch-binding protein equipped with a CBM of family 48 and a long coiled-coil motif [104] was shown to interact with Arabidopsis GBSS via a short coiled-coil motif on GBSS [95]. This interaction was required both for efficient granule binding of GBSS and for amylose synthesis.

Evidence suggests that GBSS also contributes to amylopectin synthesis. In some studies of *waxy* mutants, amylopectin structure was reported to be slightly altered [91, 105–108], whereas in other studies—sometimes on the same species—it appeared normal [109–111]. A notable exception is *C. reinhardtii* where the lack of GBSS caused an alteration in amylopectin structure: *C. reinhardtii* contains a low-molecular weight fraction of amylopectin with iodine-staining characteristics intermediate between those of amylopectin and amylose, which was missing in GBSS mutants [96]. The distinctive function of *C. reinhardtii* GBSS compared with GBSSs from vascular plants may be explained by the presence of a unique C-terminal tail and the several-fold higher specific activity [112]. Interestingly, the *C. reinhardtii* double mutant deficient in SSIII and GBSS was much more affected than each single mutant as it produced only small amounts of mostly water-soluble glucan that was almost devoid of chains with a (DP higher than 40 [97, 113]. Also early work on potato tuber starch synthesis provided evidence for a potential role for GBSS in starch synthesis. In *ssIII* repressor lines, in which GBSS was up-regulated, amylopectin had a population of extra-long chains and starch granules were altered in morphology [114]. Both traits are likely to result from the (increased) GBSS activity, as they were abolished when GBSS activity was repressed simultaneously with SSIII.

### The core amylopectin synthesis: starch synthases SSI to SSIII

Based on mutant phenotypes, each SS class appears to have a distinct role during amylopectin synthesis: put simply, SSI and SSII are thought to produce the short single-cluster-filling chains (i.e., the A- and B_1_-chains) while SSIII is proposed to synthesize longer cluster-spanning B chains [115]. In contrast, SSIV appears to be less involved in the determination of amylopectin structure but to function in starch granule initiation and the control of granule morphology [116–118]. However, in reality the situation is more complicated: there are instances of functional overlap between enzymes [119], interferences in biosynthesis by starch degradative enzymes [120–122], and of complex formation between enzymes [73, 74, 123–127]. In addition, there are sizeable gaps in our fundamental understanding of how each class fulfils its proposed role at the molecular level, as described in the following sections.

The relative contribution of each SS class varies in different tissues and between species, which is believed to account at least partially for the structural variation between starches from different sources. In the maize endosperm, SSI and SSIII constitute the major apparent soluble SS activities [128], whereas in maize leaves no SSI transcript was detected [129]. In contrast, SSII and SSIII are the major apparent soluble SS in the pea seed and potato tuber [130–132], while transcripts of potato SSI were almost exclusively detected in leaves [133]. In Arabidopsis leaves, SSI is the major soluble SS, as judged by remaining SS activity of single *ss* mutants, followed by SSIII and SSII [116, 134]. Although expressed to a reasonable level, SSIV appears to contribute only little to total SS activity [116, 135]. It is important to note, however, that estimates of the apparent contribution of an SS may be biased; suppression of SSIII, for example, is often accompanied by an upregulation of SSI and/or GBSS, meaning that the comparison of total synthase activities in the presence or absence of SSIII not only reflect the contribution of SSIII. Furthermore, SS assays themselves may preferentially measure one class over another, leading to erroneous estimations of their relative activities.

#### Starch synthase I (SSI)

Loss of SSI activity causes distinct alterations in the CLD of amylopectin, particularly concerning the A- and B_1_-chains that make up the clusters. Amylopectin from the endosperms of *ssI* mutants from rice (in a *japonica* variety, i.e., rice with inactive SSIIa and a leaky mutation in GBSS [136]) and suppressor lines in wheat [137] has more short chains of DP 6 and 7, fewer chains of DP 8–12 and more chains of around DP 18. Similar changes have also been observed in leaf starch from Arabidopsis *ssI* mutants [122, 138]. It is striking that the chains that are depleted in the mutants have the same lengths as those that are preferentially synthesized by SSI in vitro. SSI from maize, kidney bean and rice were shown to favor short chains (usually DP <10) as substrates [136, 139, 140] and SSI from Arabidopsis was more active on glycogen than on amylopectin [138]. These findings suggest that SSI elongates the short glucan chains derived from BE action (which are mostly DP 6) by a few glucan units (to a DP of around 8–10). These chains are then probably further elongated by SSII and possibly other SSs. However, since the majority of chains from BE action are still elongated in *ssI* mutants, the other SSs appear to be only partly dependent on SSI action.

It is interesting that the chains elongated in the absence of SSI appear to be elongated further, increasing the proportion of chains around DP 18 at the expense of shorter chains. Using modified glycogen substrates, it was reported that the activity of an N-terminally truncated maize SSI drastically decreases with external chain length, while its substrate binding strongly increases [139]. The authors suggested a scenario where these characteristics would make SSI stick to elongated glucan chains as an inactive enzyme, thus preventing the elongation by other synthases. Indeed, entrapment of SSI within the granule is to some extent commonly observed [128, 136, 140, 141]. Whether the extent of granule binding of SSI is really sufficient to block a significant fraction of its short chain products is unclear. Furthermore, other evidence has suggested that the granule localization of SSI is dependent on the starch binding of its interaction partner, SSII (see below).

More recent in vitro data showed that recombinant Arabidopsis SSI can actually synthesize chains up to DP 15 when incubated with maltoheptaose as primer [142] and, when assisted by a BE, was able to produce a whole spectrum of chain lengths normally present within one crystalline layer of amylopectin (i.e., A and B_1_ chains [143]). An N-terminally truncated version of barley SSI even produced chains DP >40 when maltohexose was used as primer [144]. Thus, the real reason why some of the short chains generated by SSI are not extended further in wild-type conditions remains unclear.

Arabidopsis SSI is redox-sensitive and requires reducing conditions for activity [145]. According to the crystal structure of barley SSI, it was proposed that a disulfide bridge between cysteines 126 and 506 can be formed, blocking the active site [65]. However, single mutation of each of these cysteine residues did not render SSI completely redox-insensitive, and the C506S mutant protein lost most of its activity.

#### Starch synthase II (SSII)

The effects of SSII deficiency have been characterized in potato tubers [133, 146–148], pea seeds [149], the endosperms of wheat [150], barley [151], rice (i.e., a *japonica* rice variety [152, 153]) and maize [154] and in Arabidopsis leaves [119, 122, 134]. The observed phenotypes are remarkably similar and include a distinct change in amylopectin fine structure: there is an increased abundance of chains around DP 8 and decreased abundance of those around DP 18, i.e., a shift toward shorter chain lengths. In addition, *ssII* mutant starches often have more amylose, altered granule morphology and reduced starch crystallinity. In Arabidopsis, small amounts of soluble glucan were also reported to accumulate alongside the starch [122].

Based on the alterations in CLD, it appears that SSII elongates chains of around DP 8 (the chains elongated by SSI) to lengths around DP 18. This direct interpretation is complicated by the fact that, at least in cereals, SSII interacts with SSI and class II BEs (see section “The role of complex formation and phosphorylation of starch-biosynthetic enzymes”). Hence the loss of SSII may have pleiotropic effects on these enzymes, making it difficult to assess how much of the phenotype is directly due to the absence of SSII activity. For instance, the modest increase in amylose in *ssII* mutants might be caused by altered BEII activity (see section “Specificities of BE classes”). However, it is likely that the changes in amylopectin fine structure are caused by the lack of SSII activity. First, when a recombinant rice SSII was incubated with amylopectin from a rice *ssII* mutant, it specifically elongated the aberrantly short chains so that the modified glucan now appeared more wild-type like [155]. Second, the loss of SSI activity in an *ssII* mutant background caused *ssI*-typical alterations, indicating that SSI was still active [122, 134, 136]. Third, the changes in amylopectin CLD are similar in dicots plants where there is no evidence for the formation of SSII-containing complexes.

#### Starch synthase III (SSIII)

Compared with SSI and SSII, the function of SSIII is less clear. Its suggested functions include the synthesis of long B chains, the elongation of cluster-filling chains (partly redundant function with SSII) and the regulation of other starch-biosynthetic enzymes. Furthermore, SSIII is important for the initiation of starch granules, at least in the absence of SSIV. Consistent with the view of a versatile role, SSIII appears as a major soluble SS activity in all plants and tissues that have been analyzed to date. It also harbors the longest N-terminal extension among all SS, which carries starch-binding domains and predicted coiled-coil domains (Fig. 2).

Probably the best characterized function of SSIII lies in the synthesis of long, cluster-spanning B chains (i.e. B_2_, B_3_ etc.). Fewer of these chains were observed in *ssIII* mutant starches from potato tubers [148], endosperms of maize [109, 156] and rice [157], and in *C. reinhardtii* [97]. Of the *ssIII* mutants characterized, only the Arabidopsis *ssIII* mutant [158] and the barley *amo1* mutant do not display this phenotype (note that the *amo1* mutation does not to abolish SSIII activity, however [159]).

Alterations in the short chain profile of amylopectin from *ssIII* mutants [147, 148, 157, 158, 160] indicate that SSIII is also involved in the synthesis of short A and B chains. These changes are subtle when compared to those caused by the lack SSI or SSII. In the absence of SSII, however, additional loss of SSIII significantly enhances the mutant phenotype in rice grains [80], potato tubers [147, 148] and Arabidopsis leaves [119], suggesting partially redundant functions between these two SSs. The *ssII/ssIII* double mutant of Arabidopsis produced tiny amounts of glucans with drastically shortened chains, a fraction of which was water soluble [119, 122]. In potato tubers, the combined repression of SSII, SSIII and GBSS resulted in amylose-free starch with short-chained amylopectin, gels of which did not undergo retrogradation upon repeated freeze–thaw cycles—a preferred characteristic for the food industry [161].

Arabidopsis *ssIII* mutants were reported to have increased total soluble SS activity [158] and to produce more starch during the day [117, 158] in the leaf, leading to the suggestion that it has a negative regulatory function on other starch-biosynthetic enzymes. Other studies, however, reported slightly reduced soluble SS activity [117, 134] and starch levels [122, 134]. An increase in SSI and/or GBSS levels was observed in *ssIII* endosperms from maize [128] and rice [157] and in *C. reinhardtii* [97]. It is therefore possible that some of the alterations in amylopectin fine structure are caused by increased SSI levels. Furthermore, the observed increase in amylose content in *ssIII* endosperm starches from maize [156, 162] and rice [111, 157] may be due to enhanced GBSS activity. The increased action of GBSS is also likely to be the cause for other phenotypes associated with a deficiency in SSIII such as an increased number of extra-long amylopectin chains and fissured granules [114, 157]; repression of GBSS in addition to SSIII in potato tubers suppressed these aspects of the *ssIII* phenotype [114].

### The initiation of starch granule formation: starch synthase IV (SSIV) and other factors

To date, mutants with reduced SSIV activity were described only in rice [80, 163] and Arabidopsis [116–118]. Rice has two SSIV isoforms: *Os*SSIVa, which is expressed only little in leaves and endosperm, and *Os*SSIVb, which is generally higher expressed [79, 80]. Neither single repressors of *Os*SSIVa or *Os*SSIVb [80], nor null mutants of *Os*SSIVb (in a *japonica* variety; [163]) show marked alterations in starch content or structure in the seed endosperm. In contrast, null mutants of the single SSIV isoform in Arabidopsis show alterations in diurnal leaf starch content, having less starch at the end of the day and more at the end of the night compared with wild-type plants, despite having a normal amylopectin fine structure [116]. The Arabidopsis *ssIV* mutant also has remarkable alterations in the number and shape of starch granules: instead of around six discoid granules [164], chloroplasts from *ssIV* mutants have zero, one or two granules, which are enlarged and spherical with a less electron-dense center [116, 118]. In young *ssIV* leaves, the chloroplasts were starch free [118]. Overexpression of *At*SSIV in Arabidopsis increased total leaf starch content, although an increase in starch granule number was not reported [165].

Overall, Arabidopsis SSIV clearly has a unique function regarding the initiation and morphology of starch granules, and the degree of starch accumulation [116, 118]. Interestingly, the levels of ADPglucose (the substrate for SS) in *ssIV* mutants are increased over 50-fold suggesting that its consumption is strongly limited [118]. This is especially likely to be the case in starch-free chloroplasts where the remaining SS isoforms lack glucan substrate. In contrast, Arabidopsis plants having SSIV as sole soluble SS (i.e. *ssI/ssII/ssIII* mutants) produce similar numbers of granules per chloroplast as the wild type, despite having only little, aberrant starch overall [117]. It will be interesting to see whether there are similar impacts on leaf starch in the rice *ssIVb* mutant and repressor lines.

Analyses of multiple SS mutants in Arabidopsis indicate that SSIII (but not SSI and SSII) is required to achieve some starch synthesis in the absence of SSIV: Arabidopsis *ssIII/ssIV* mutants almost completely fail to synthesize starch and display chlorosis and stunted growth [37, 117]. Despite a strongly reduced rate of photosynthesis, these mutants still accumulate around 100 times more ADPglucose, unbalancing the adenylate pool and possibly explaining the pleiotropic defects observed [37]. The consequence of losing SSIIIa and SSIVb in *japonica* rice was quite different, although still striking. The *ssIIIa/ssIVb* double mutant was still able to produce substantial amounts of endosperm starch, but accumulated spherical and loose granules instead of the polyhedral compound-type starch granules normally observed [163]. This phenotype was accompanied by an uneven amyloplast surface and a modified galactolipid composition of membranes. Alterations in the latter have previously been implicated in the transition from simple- to compound-type granules in the *opaque5* maize mutant [166]. Toyosawa and colleagues hypothesized that SSIVb may be important for the structural integrity of as-yet uncharacterized, membrane-containing septum-like structures [163] that may form a mold within amyloplasts for casting starch into the polyhedral shape [167]. Possible reasons for the phenotypic differences between *ssIIIa/ssIVb* rice and *ssIII/ssIV* Arabidopsis include the presence of other SSIII and SSIV isoforms in the rice endosperm and/or mechanistic differences in storage and transitory starch granule formation.

The function of SSIV and the mechanisms for determining granule number and shape remain enigmatic. Glycogen biosynthesis in other organisms begins with self-glucosylation either of glycogen synthases (in bacteria; [168]) or of specialized proteins named glycogenins (in eukaryotic cells; [169, 170]). Introducing the self-priming *A. tumefaciens* glycogen synthase into Arabidopsis *ssIII/ssIV* double mutants indeed restored initiation of multiple starch granules per chloroplast, but the granules’ morphology was still like those found in the *ssIV* [118]. Attempts to confirm such a self-priming activity in recombinant *At*SSIV (and *At*SSIII) were not successful [117], although *At*SSIII was found to catalyze unprimed glucan formation in the presence of ADPglucose [117]. An autoglucosylation function may not be required to explain the initiation of starch polymers in photoautotrophic tissues if ideas about de novo synthesis of maltose during photosynthesis prove to be true [171, 172]: all four soluble SSs from Arabidopsis are able to elongate maltose and longer malto-oligosaccharides (but not glucose) [142].

Arabidopsis SSIV was reported to interact with two plastoglobule-associated proteins, the fibrillins FBN1a and FBN1b [75]. This interaction was dependent on the presence of the non-catalytic N-terminus of SSIV predicted to contain coiled-coil motifs (Fig. 2). Localization studies of SSIV report it to be a stromal protein loosely targeted to distinct spots within the chloroplast—initially proposed to be the edges of granules [117]. However, more recently, it was reported to be associated to thylakoid membranes [75]. In the rice endosperm, SSIVb also had a sub-plastidial localization, being predominantly distributed in the space between starch granules, although distinct spots were rarely observed [163]. Given its hypothesized role in determining granule number and morphology, the specific positioning of SSIV within the plastid may well turn out to be a factor of critical importance. In turn, understanding the factors that influence its localization—to the granule or to membranes—is likely to give completely new insight into the control of starch formation.

Additional factors have been implicated in the control of starch granule number. A number of proteins that self-glucosylate using UDPglucose were described over the years (e.g. [173–175]), yet none were reported to have plastidial targeting signals, but were mostly implicated in cell-wall biosynthesis [176]. Proteins homologous to glycogenins were reported in Arabidopsis, and suppression of one containing a predicted chloroplast-targeting signal appeared to reduce starch accumulation [177]. This observation, based on iodine staining, has not been substantiated. Interestingly, mutant phenotypes have also suggested the involvement of α-glucan phosphorylase and isoamylase DBEs as influencing starch granule number (described in later sections). It is also interesting to note that one of the proteins implicated in the reversible glucan phosphorylation, Starch Excess4 (SEX4)—whose activity is required for starch degradation at night—also has a profound impact on starch granule morphology, as its loss resulted in very large, round granules [26]. This phenotype has not been reported for other mutants affected in starch degradation and its basis has yet to be investigated.

### Classes and domain structure of branching enzymes (BE)

**Fig. 3:**
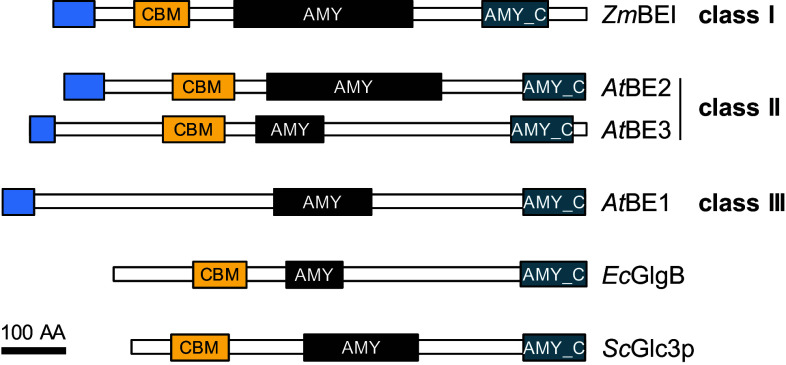
The domain structure of branching enzymes (BE). BE classes can be distinguished by sequence but share a common domain structure. Depicted are plastidial transit peptides (N-terminal *blue boxes*), carbohydrate-binding modules of family 48 (*orange boxes*, CBM), catalytic α-amylase family domains (*black boxes*, AMY) and the all-β domains typically found in the C terminus of α-amylases family members (*blue boxes*, AMY_C). Domain predictions were obtained as described in Fig. 2. No coiled-coil domains were predicted with the settings described in Fig. 2. The domain structure is conserved between classes and orthologs, although the length of the predicted catalytic α-amylase family domain varied. As Arabidopsis does not have a class I BE, maize BEI (*Zm*BEI) is included for comparison, as are glycogen BEs from budding yeast (*Sc*Glc3p) and *E. coli* (*Ec*GlgB). *At*BE1 is a putative BE of class III which lacks the CBM48 and has not yet been shown to display branching activity. *Bar* 100 amino acids (AA)

Starch-branching enzymes (E.C. 2.4.1.18) belong to the α-amylase superfamily of enzymes (also termed glycoside hydrolase family 13; CAZy: [59]; recently reviewed [178]. They cleave an α-1,4-glucan chain and transfer the cleaved portion to the C6 position of a glucose unit from the same or another chain, creating an α-1,6 linked branch. In doing so, BEs generate additional substrates for the SSs (i.e., non-reducing ends of chains). BEs share a common three-domain structure: an N-terminal domain containing the CBM of family 48, a central catalytic α-amylase domain characteristic for GH13 family members and a C-terminal domain present in several α-amylases (Fig. 3).

According to sequence similarities, BEs are separated into class I (or family B) and class II (family A) enzymes. Class I enzymes generally occur as a single isoform in planta, with the exception of Arabidopsis, which does not contain a class I BE [179]. Class II is represented by a single gene in potato and pea, but two isoforms—BEIIa and BEIIb—occur in the cereals. These have distinct expression patterns: in maize, rice and barley, the expression of BEIIb is restricted to the grain, while BEIIa is found in all tissues but at often lower levels [180–183]. Arabidopsis also has two type II BEs (BE2 and BE3), both of which are expressed in leaves and appear largely functionally redundant [179].

Protein-sequence homology revealed a putative third class of BE. Genes belonging to this class III were found in Arabidopsis (BE1; [179]), rice, poplar and maize (BEIII; [78, 184]. Despite the high homology within this class (around 60 %), the proteins share only around 30 % identity with BEs from class I and II [184]. To date, functional analysis on this putative BE was only done in Arabidopsis, where knock-out mutants were first reported have a wild-type phenotype [179], then to be inviable [185]. Branching activity was not reported by either group. Further work is required to reveal the catalytic activity and biological function of these proteins.

### Specificities of branching enzyme (BE) classes

The specific placement of branches by BEs is believed to be a major determinant of the cluster structure of starch (i.e., the enrichment of branches in certain, regularly spaced regions). However, due to our limitations in assessing the actual distribution of branch points, investigations on the specificity of BE isoforms often focused more on the lengths of transferred chains rather than their placement, primarily by comparing the CLDs of glucans before and after the in vitro modification by a BE. Accordingly, class I BEs tend to transfer slightly longer chains than BEII and are more active towards amylose, whereas class II BEs typically prefer amylopectin [186–190]. Further, BEI from rice modified a phosphorylase-limit amylopectin, suggesting that this BE has can transfer already-branched chains [190, 191]. Obtaining the phosphorylase-limit CLDs after BE action allows modifications on the inner branching structures to also be analyzed [191]. This revealed that all rice BEs produce a relatively broad distribution of distances between new and old branches [191]. In contrast, the glycogen BE from *E. coli* (*Ec*GlgB), while transferring chains of similar length to plant BEIIs, differed in that it preferentially placed new branches only three glucose units away from an existing branch point [191]. This structural trait was also observed when *Ec*GlgB was expressed in an Arabidopsis line deficient in its endogenous BEs (*be2/be3* double mutants) [192]. Nevertheless, *Ec*GlgB restored the synthesis of insoluble glucans [192, 193], albeit in reduced amounts and in the form of irregular particles.

Mutants of BEI have been analyzed in maize [194, 195], rice [6, 196] and wheat [197] endosperms, and the gene repressed in potato tubers [198]. In all cases, deletion or reduction of BEI resulted in only minor changes on starch structure/amount at best, such that its function in vivo remains unclear. Reduction of most or all BEII activity, however, caused marked alterations in starch and the well-known *amylose*-*extender* (*ae*) phenotype. This phenotype was observed in *beIIb* mutants of maize [199] and rice [200], in *beIIa* mutants of durum wheat [201], in mutants of BEII in pea (yielding the wrinkled-seed mutants studied by Gregor Mendel [202, 203]) and in double repressor lines of BEIIa and BEIIb in wheat and barley [204, 205]. The *ae* phenotype is characterized by altered amylopectin structure with longer external and internal chains and, in some cases, increased levels of amylose [6, 106, 202, 206–208]. Due to its special swelling and gelatinization properties and reduced digestibility in the digestive tract, *ae* starch is of special interest both for industry and human health [11, 30]. In rice grains, expression levels of BEIIb correlated with amylopectin structure, and increasing repression of BEIIb was accompanied by a change from A to C to B polymorph [207, 209]. Upon overexpression of BEIIb, glucans with lower crystallinity than normal were observed and soluble glucans also accumulated [209].

In potato tubers, repression of BEII led to amylopectin with longer chains [210], but a high amylose content was only seen upon suppression of both class I and II BE genes [211]. This high-amylose starch also had high phosphate levels—another feature of considerable industrial interest. However, the overall starch yield was severely reduced [211]. Overexpression of BEII in potato was recently reported to increase the abundance of short amylopectin chains (mostly DP 6), lower the gelatinization temperature of the starch and to reduce the phosphate content [212].

Some mutations in BE affect leaf starch metabolism. Maize *beIIa* mutants have normal endosperm starch, but mostly replace leaf starch by a glucan of low molecular weight and a low degree of branching [213, 214]. Although BEIIa is the predominant BE in maize leaves, transcripts of BEI were also found in the leaf [214], presumably accounting for the residual glucan synthesis. In Arabidopsis, mutants disrupted in both class II BEs (BE2 and BE3) exhibit a complete loss of BE activity and fail to produce starch. Instead they accumulate high amounts of maltose [179]. It was hypothesized that this water-soluble glucan was generated during the simultaneous synthesis of linear chains by SSs and degradation by the plastidial α-amylase 3 (AMY3) and/or β-amylases.

Collectively, the numerous studies of BEs show that, as expected, branching activity is absolutely required for amylopectin synthesis. They also show that BE specificities differ and that they significantly influence the final glucan structure. Furthermore, their activity relative to that of SSs is important, which is illustrated by the change in glucan structure when expressed to differing extents [209, 212]. Nevertheless, BEs are not the sole determinants for the synthesis of crystallization-competent glucans, as demonstrated by the ability of *E. coli* glycogen BE to restore some insoluble glucan synthesis [192, 193]. It should also be kept in mind that BEs form complexes with other starch-biosynthetic enzymes, which might influence their functions (described below).

### Debranching enzymes (DBEs) and the trimming of glucans

**Fig. 4:**
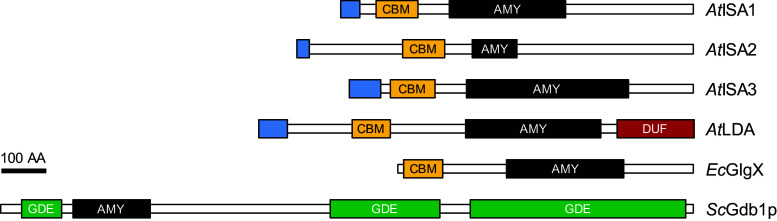
The domain structure of debranching enzymes. The Arabidopsis debranching enzymes (*At*ISA1, *At*ISA2, *At*ISA3, *At*LDA) and *E. coli* debranching enzyme (*Ec*GlgX) share the domain structure with BEs. The indirect debranching enzyme from budding yeast (*Sc*Gdb1p) is shown for comparison. Depicted are the plastidial transit peptides (N-terminal *blue boxes*), carbohydrate-binding modules of family 48 (*orange boxes*, CBM), catalytic α-amylase family domains (*black boxes*, AMY) and a Pfam domain of unknown function, DUF3372 (*red box*, DUF). Domain predictions were obtained as described in Fig. 2. No coiled-coil domains were predicted with the settings described in Fig. 2. Where full-length sequences could be obtained, the domain structures of classes were conserved among orthologs in various species. *Sc*Gdb1p contains three Pfam glycogen-debranching enzyme (GDE) domains (hGDE_N, hGDE_central and GDE_C, from N to C terminus). These are characteristic for eukaryotic GDEs, which combine oligo-α-1,4 → α-1,4-glucanotransferase (EC 2.4.1.25) and α-1,6-glucosidase (EC 3.2.1.33) within a single enzyme and debranch glycogen in a two-step mechanism. *Bar* 100 amino acids (AA)

Plant DBEs hydrolyze α-1,6-linkages and release linear chains. They belong to the glycoside hydrolase family 13 (CAZy: [59]) and share the central α-amylase domain and a starch-binding domain with BEs (Figs. 3, 4). They can be further divided into two types: isoamylases (ISAs; E.C. 3.2.1.68) and limit-dextrinase (LDA; E.C. 3.2.1.41). The two types can be distinguished by protein sequences and substrate specificity, as only LDAs can efficiently degrade pullulan [215, 216], a yeast-derived glucan consisting of α-1,6-linked maltotriosyl units (hence the enzyme’s alternative name pullulanase or PU1). Plant genomes encode three classes of isoamylase—ISA1, ISA2 and ISA3—and one LDA.

ISA1 and ISA2 are involved in debranching during the synthesis of amylopectin and are thus in the focus here. They act together as a heteromultimeric enzyme, and ISA1 additionally forms active homomultimers in some species (described below)—hereafter we use “ISA” to refer to both homomeric and heteromeric activities. In contrast, ISA3 and LDA primarily debranch starch during its degradation and mutants of these enzymes often have *starch*-*excess* (*sex*) phenotypes [217–220]. Nevertheless, ISA3 and LDA do influence the glucan made in the absence of ISA, although they cannot replace its specific function [121, 218, 221–223].

Mutants lacking ISA partly replace starch by a water-soluble polysaccharide in the endosperms of maize [224, 225], rice [226] and barley [227], in potato tubers [228], Arabidopsis leaves [120, 218] and in *C. reinhardtii* [229]. In terms of branching level, wavelength of maximum absorption after complexion with iodine and molecular weight, this water-soluble glucan appears reminiscent of glycogen and was hence called phytoglycogen [230]. In most cases, insoluble starch, albeit with small structural alterations, is still made. Only in *C. reinhardtii* and in *japonica* rice (i.e., rice with an *ssII* background), do *isa1* mutants completely fail to produce starch [226, 229]; in the latter, this is dependent on the mutated *ssII* allele [231].

**Fig. 5:**
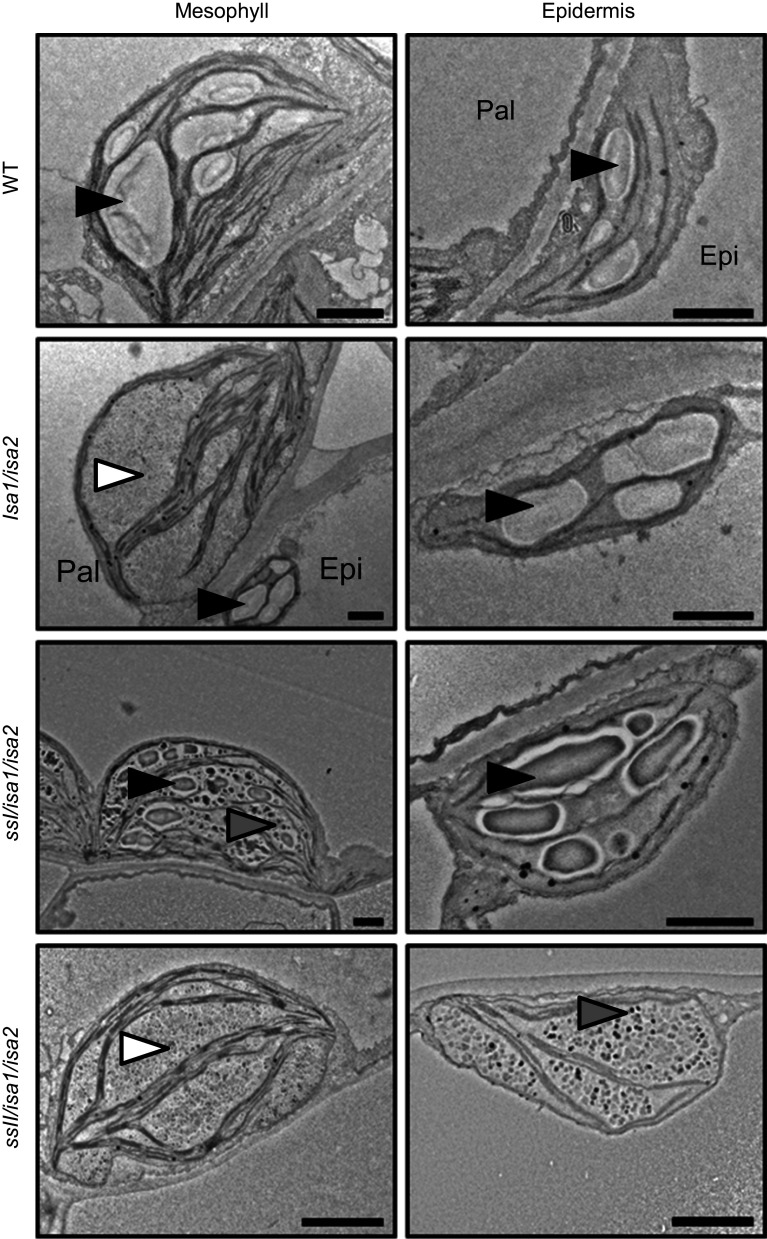
Modulation of the *isa* mutant phenotype by starch synthases. Transmission electron micrographs of Arabidopsis chloroplasts at the end of day. Wild-type plants (WT) contain starch granules (*black arrowheads*) in the mesophyll and epidermis. The *isa1/isa2* mutant accumulates predominantly phytoglycogen (*white arrowheads*) in the mesophyll, but still makes starch in the epidermal cells. Additional loss of SSI restores the formation of starch granules in the mesophyll, most likely due to a relative increase in the proportion of longer, cluster-filling chains that promote crystallization, despite aberrant branching. In contrast, additional loss of SSII completely abolishes the synthesis of insoluble glucans, even in the epidermal cells. This probably stems from the relative increase in shorter chains in the absence of SSII, impairing the formation of secondary and tertiary structures. *Epi* epidermal cell, *Pal* palisade cell, *Bars* 1 µm. Adapted from [122], Copyright American Society of Plant Biologists (http://www.plantphysiol.org)

A common model to explain the accumulation of phytoglycogen is the “trimming” model [232]. According to this model, ISA removes excess branches from a newly created amorphous zone in “pre-amylopectin” so that only the appropriate chains are elongated by SSs. In the absence of ISA, the high number of branches would result in steric limitations, abolishing regular structures and the synthesis of further lamellae. Consistent with this, Arabidopsis phytoglycogen is indeed enriched in branches separated by short distances [120]. However, while this model regards debranching by ISA as a mandatory step, careful observation of mutants lacking the enzyme suggest that this is not strictly essential for making a crystallization-competent glucan: Not only do most ISA-deficient mutants make some starch, often both starch and phytoglycogen are made within the same plastid and some cell types/tissues appear little affected (e.g. bundle-sheath cells from Arabidopsis and maize leaves [120, 233, 234]). Streb et al. showed that Arabidopsis leaf starch is completely replaced by phytoglycogen in the *isa1/isa2/isa3/lda* mutant lacking all DBEs [121]. This suggested some functional overlap between the DBEs—a result consistent with previous observations in Arabidopsis [218, 223], maize [217] and rice [221, 222]. However, the additional mutation of the starch degrading enzymes α-amylase 3 (AMY3) in the debranching mutant background partly restored starch granule synthesis, showing that debranching, while part of the biosynthetic process, is not an absolute requirement [121]. This result was in-line with the earlier observation that phytoglycogen is prone to degrading enzymes [120], and suggested that the modifications made by degradative enzymes also served to prevent nascent molecules from forming insoluble starch granules. Recently, genetic analyses in Arabidopsis [122] and rice [231] suggested that the lengths of the cluster-filling chains also influence whether insoluble glucans are made in the absence of ISA; relative increases in longer chains promoted insoluble glucan formation, while enrichment of shorter chains abolished them altogether. For instance, *ssI/isa* double mutants have longer A and B_1_ chains and produce significantly more starch than *isa* single mutants [122] (Fig. 5). It thus seems that debranching facilitates or accelerates the crystallization of glucans rather than formally enabling it. The crystallization itself in solid granules may in turn protect the glucans from premature degradation by amylases [120, 121].

Interestingly, recent studies suggest that ISA itself can act in a degradative fashion under some circumstances. First, expression of *At*ISA1–*At*ISA2 in *E. coli* [235] and *S. cerevisiae* (B.P. and S.C.Z., unpublished data) impeded the accumulation of glycogen instead of yielding a more amylopectin-like glucan. Second, far fewer glucans were observed in the Arabidopsis *ssII/ssIII* double mutant compared with the *ssII/ssIII/isa* triple mutant [122]. Notably, the glucans made in *ssII/ssIII* plants showed structural similarities to glycogen in that they had much shorter chains than wild-type amylopectin and were partly found in the water-soluble fraction [122]. These data indicate that debranching by ISA to promote crystallization requires the appropriate glucan substrate—a pre-amylopectin with adequate chain lengths and/or branching pattern. If the glucan structure is incompatible with (rapid) crystallization upon debranching (e.g. because of insufficiently long chains), the ongoing accessibility may cause excess debranching and ultimately glucan degradation.

A purely degradative action of ISA may also provide an explanation for the formation of numerous small granules/particles in *isa* mutants [227, 228, 236]. In Arabidopsis, this phenotype has been suggested to be a secondary effect resulting from the enhanced α- and β-amylolytic degradation of soluble glucans: this could yield small soluble oligosaccharides that could serve as primers for glucan synthesis or as nucleation sites for starch granules [121]. However, small branched oligosaccharides, possibly representing the earliest stage of normal glucan synthesis, would also be excellent targets for debranching by ISA, as mentioned above. Debranching and clearance of such glucans could then limit the pool of potential nucleation sites for starch granules.

#### Distinct functions of isoamylase homo- and heteromultimers

Depending on the species, ISA1 either acts exclusively in heteromultimeric complexes formed between ISA1 and ISA2, or both as heteromeric and homomeric complexes. The Arabidopsis *isa1*, *isa2* and *isa1/isa2* mutants show identical phenotypes [120]. Moreover, *At*ISA1 and *At*ISA2 have a high native molecular mass, interact with each other and are stable only in the presence of their partner [120, 235]. Catalytic activity was only observed when both *At*ISA1 and *At*ISA2 were present [235, 237]. These data strongly suggest that ISA1 and ISA2 form an obligate heteromultimeric enzyme. ISA2 carries substitutions in amino acid residues important for catalysis, probably rendering it non-catalytic. ISA1 is therefore likely to be the catalytic subunit, with ISA2 being required for the enzyme’s stability, and possibly for its specificity and/or regulatory properties [235, 238, 239]. Similarly, in potato, only a heteromultimeric complex of ISA1 and ISA2 was found [240], although potato ISA1 was active on its own when recombinantly expressed as an S-tagged protein in *E. coli* [238].

In other species it is clear that ISA1 also acts as a homomultimer. One or several homomeric complexes of ISA1 were found in addition to at least one ISA1/ISA2 complex in rice and maize grains and in *C. reinhardtii* [240–242]. In a recent crystal structure, *C. reinhardtii* ISA1 had an elongated structure and paired with another ISA1 in a tail-to-tail fashion, although the existence of higher multimers could not be excluded [242]. Maize and rice *isa2* mutants, which have only the ISA1 homomer, had similar endosperm starch synthesis as wild-type plants, suggesting that the ISA1 homomers are sufficient [241, 243]. However, in an *ssIII* mutant background, loss of ISA2 resulted in the accumulation of phytoglycogen in maize kernels [160]—an intriguing, albeit unexplained observation. The ISA1/ISA2 heteromultimer was also reported to be more thermostable and have a higher affinity for phytoglycogen than ISA1 alone [240]. Thus, both the catalytic properties and stability of ISA1 seem to be influenced by ISA2, and both homomeric and heteromeric ISAs may be required for optimal amylopectin synthesis, at least in some conditions.

### The role of complex formation and phosphorylation of starch-biosynthetic enzymes

For the past decade there has been mounting evidence for the formation of complexes between starch-biosynthetic enzymes and for the phosphorylation of these enzymes. Recently, two additional complexes were identified which are distinct as they involve proteins without apparent catalytic functions (see section “Starch-binding proteins and other novel proteins”). In an early study investigating this topic, co-immunoprecipitation indicated that BEIIb interacts with BEI and with starch phosphorylase in the wheat endosperm [123]. Treating the protein extract with alkaline phosphatase abolished these interactions, while incubation with ATP had the opposite effect. Thus, phosphorylation was proposed to be a prerequisite for complex formation, consistent with the observation that all wheat BEs can be phosphorylated [123]. Later, size-exclusion chromatography (SEC) of maize endosperm amyloplast extracts showed that SSII, SSIII, BEIIa, BEIIb occur to varying extents as non-monomeric forms. Pair-wise interaction tests via immunoprecipitation, affinity purification and yeast two-hybrid suggested a phosphorylation-dependent complex of SSIII, SSII, BEIIa, BEIIb (and possibly SSI) and a second, similar complex without SSIII [73, 74]. Meanwhile, Tetlow and coworkers reported other phosphorylation-dependent complexes in the wheat endosperm, among them one between SSI, SSII and BEIIa or BEIIb [124]. This observation was also based on pair-wise co-immunoprecipitation, similar elution profiles obtained using SEC, and subsequent western-blotting of cross-linked fractions.

The association of SSI, SSII and a class II BE appears to be conserved at least within the cereal endosperm, as evidence for it was also found in maize [125], barley [126] and in rice [127]. In wheat and barley seeds, where BEIIa and BEIIb have redundant functions, this complex contains either of them and, in barley, possibly both [124, 126]. In the endosperm of maize and rice, where BEIIb is the dominant BE, BEIIa normally is not associated with the complex [125, 127].

Knowing about the existence of these protein complexes inevitably raises the question of their significance. In particular, it is important to understand how the functions of enzymes in complex differ when compared to those of their monomeric forms. This information will strongly affect the interpretation of mutant phenotypes and therefore our understanding of the role of individual enzymes: if the lack of a single enzyme abolished the formation of a whole complex and altered the activities of the remaining now solely monomeric or partially complexed enzymes, the resulting phenotype would not only result from the missing enzyme activity but also from the alteration of the biosynthetic machinery as a whole.

In *ssII* mutants, SSI and the interacting BEII(s) consistently appear less in the granule-bound fraction [150, 151, 244]. This was also observed in a starch-binding mutant of SSII [245]. Furthermore, in the absence of BEIIb in maize, a modified complex is formed in the endosperm: SSI and SSII now interacted with BEI, BEIIa and phosphorylase instead [125, 246]. Similarly, repressed BEIIa or BEIIb was replaced by BEI and phosphorylase in the complex in barley [126]. In both cases, protein replacement was reflected in the profile of granule-bound proteins: the newly complexed enzymes became granule bound, together with their complex partners and GBSS [125, 126]. This suggests that proteins in the complex have a higher affinity for starch. Nevertheless, it is worth noting that both BEIIb and SSI can bind starch on their own [139, 245, 246] and it remains unclear whether a tight association or even entrapment is required for optimal activity.

A potential biological function for complex formation could be to channel substrates from one enzyme to another—a newly created branch resulting from BE action could be directly elongated first by SSI and then by SSII, thereby increasing the overall efficiency of the process. It is also possible that the complex confers enzymatic specificity; for instance, steric limitations of the whole complex could define the length of a chain that is transferred by a BE or where exactly a new branch is placed. Tetlow and Emes [115] further suggested that complex formation could regulate enzyme activity allosterically and/or could protect a growing glucan from degrading enzymes. Still, none of these hypotheses has yet been tested.

Similarly, the role of phosphorylation of BEs and some SSs [28] remains enigmatic. Besides promoting complex formation, phosphorylation also increased the activity of stromal BEIIa and BEIIb in wheat [123]. Mutating conserved phosphorylation sites of maize BEIIb to serine drastically reduced the activity of the recombinant protein in vitro [247]. Since this activation happened in the absence of its interaction partners, it shows that phosphorylation does not necessarily increase BEIIb activity through complex formation. The authors furthermore presented evidence for the involvement of Ca^2+^-dependent protein kinases in phosphorylation of BEIIb.

It is clear that there will be much need for detailed biochemical and structural analyses of these protein complexes in the future. Particularly the creation of mutant proteins that are still active, but unable to interact with its enzymatic partners, would help in establishing the importance of the complexes.

### Additional factors with putative implications in starch synthesis

#### The reversible phosphorylation of glucans

The reversible phosphorylation of starch is best characterized in Arabidopsis where it is pivotal for efficient degradation. It is believed that phosphorylation of glucosyl units at the C6 or C3 positions (exerted by glucan, water dikinase, GWD, or phosphoglucan, water dikinase, PWD, respectively [248, 249]) disrupts and destabilizes the helical structures formed by glucan chains, rendering them accessible to degrading enzymes, in particular β-amylases [250]. Since exo-acting β-amylases cannot degrade past phosphate groups, these need to be hydrolyzed concomitantly by the phosphoglucan phosphatases SEX4 (hydrolyzing at C6 and C3 [251]) and Like-SEX4 2 (LSF2; hydrolyzing at C3 [252], recently reviewed in [253]).

However, glucan phosphorylation is not confined to the dark period. Arabidopsis leaf starch made during a single photoperiod is C6- and C3-phosphorylated, showing that both GWD and PWD are active during the day. Furthermore, *lsf2* mutants have increased levels of C3 phosphorylation at the end of the day, even though these mutants almost completely degrade their starch at night. This suggests that phosphate groups introduced during the day are normally also being removed by LSF2 [252]. In addition, it has been shown that the amount of C6-bound phosphate in Arabidopsis starch at the end of the day is variable and correlates positively with photoperiod [254]. Incubating potato tuber disks with glucose and radiolabeled orthophosphate resulted in radiolabeled phosphate in starch, also indicating that phosphate is incorporated during net synthesis of storage starch [255].

The relevance of phosphorylation during synthesis is not clear yet. Starches from Arabidopsis *gwd* mutants, which are essentially phosphate free, have a normal internal structure but differ in their granular surface, which is irregular and displays shorter chains [256]. These differences may, however, be secondary effects from incomplete starch degradation during the night and the subsequent synthesis of glucans on top of these altered granular surfaces. Arabidopsis *gwd* mutants also have only little net starch synthesis during the day. This could mean that lack of GWD inhibits starch synthesis generally [257]. However, by silencing GWD during an extended night, Skeffington and colleagues obtained plants without remaining starch and GWD, and these synthesized wild-type amounts of starch in the following day [257]. It, thus, appears unlikely that GWD is directly required during the process of starch synthesis. Rather, phosphorylation of glucans by GWD for efficient glucan degradation during the night may be important subsequent starch synthesis: either directly by providing proper starting material or indirectly by affecting carbon partitioning (*gwd* mutants starve during the night, which can influence the utilization of photoassimilates the following day).

#### α-Glucan phosphorylase—phosphorolysis or synthesis?

α-Glucan phosphorylase (EC 2.4.1.1) catalyzes the reversible reaction (α-1,4-linked glucose)_*n*_ + glucose-1-phosphate ⟷ (α-1,4-linked glucose)_*n*+1_ + orthophosphate. Higher plants have two classes of phosphorylase: a plastidial and a cytosolic one. The plastidial class, named Pho1, PhoL (because of its low affinity towards glycogen) or PHS1, carries a 78-amino acid insertion near the glucan-binding site and shows highest affinity towards linear glucans, especially malto-oligosaccharides. By contrast, the cytosolic class, named Pho2, PhoH or PHS2, prefers branched substrates such as glycogen [258, 259].

The function of Pho1 is ambiguous, to the extent that it is not even clear whether it acts in a synthetic or phosphorolytic (i.e., degrading) way in vivo. The plastidial concentration of orthophosphate typically exceeds that of glucose-1-phosphate manifold [260], which would favor phosphorolysis in vivo. Nevertheless, rice Pho1 was shown to catalyze chain elongation as well as chain shortening under comparable concentrations in vitro [261, 262]. Rice mutants of Pho1 produce endosperm starch with slightly shorter chains, lower gelatinization temperature and altered granule shape, although this phenotype was not fully penetrant [261]. An additional phenotype was observed with the majority of the grains being shriveled and containing little starch when plants were grown at lower temperatures (20 °C) [261]. It was suggested that, under these conditions, Pho1 may work in a biosynthetic direction helping to initiate starch synthesis by synthesizing malto-oligosaccharide primers for SSs, explaining the conditional loss of starch in the mutants. However, repression of Pho1 in barley [263] had no apparent effect on starch granule initiation, although granule number was not assessed.

Nevertheless, the gene expression and/or enzyme activity of Pho1 correlates with that from known starch-biosynthetic genes in potato tubers [264] and the endosperms of maize, wheat and barley [263, 265, 266], consistent with a role in biosynthesis. If acting in the phosphorolytic direction, Pho1 could help degrade malto-oligosaccharides created during the glucan trimming by isoamylase, yielding glucose-1-phosphate for subsequent re-conversion to ADPglucose [267]. Given that Pho1 was found to interact with BEIIb and BEI in wild-type wheat and maize amyloplasts [123, 268] and with SSI, SSIIa and BEI in *beII* mutants of maize and barley [125, 126], Pho1 may also act indirectly by modifying their enzyme activities or directly on their products.

In *C. reinhardtii*—which differs from vascular plants in having two plastidial isoforms, PhoA and PhoB—loss of PhoB resulted in reduced starch content. The remaining starch furthermore had a higher relative amylose content and structural modifications [267]. Still, this phenotype was confined to conditions of nitrogen starvation where flux into starch is highest. PhoB also is more similar to the cytosolic than the plastidial class of vascular plants as it lacks the typical sequence insertion and has a low affinity for malto-oligosaccharides [267].

There has also been speculation that PhoI may be involved in starch degradation. However, no significant influence on leaf starch content was noted upon repression of the leaf isoform of Pho1 in potato [269] or in the *pho1* mutant of Arabidopsis [270–272]. Although these reports point against an essential role during degradation of transitory starch, local accumulation of starch was observed around leaf lesions, leading to the speculation about a specific role for phosphorylase in providing respiratory substrates during stress responses [270, 273]. More recently, genetic evidence for the participation of Arabidopsis Pho1 in starch degradation has been obtained from Pho1 deficiency in the background of *ssIV* mutants and those impaired in maltose metabolism [271, 272].

#### Starch-binding proteins and other novel proteins

In recent years, several novel proteins have been identified that influence starch structure and/or amount. In some cases, the impact on starch may be an indirect one whereas in others, the proteins may be part of a hitherto undiscovered aspect of the starch-biosynthetic process. In either case, the analysis of the underlying mechanisms has the potential to provide new insights into facets of starch biosynthesis that go beyond the core enzyme machinery, such as the regulation of starch synthesis or the influence of the plastidial environment on granule formation and morphology.

Peng and colleagues described the rice Floury Endosperm6 (FLO6), a plastidial protein with a CBM48 and the capacity to bind starch [274]. Grains deficient in FLO6 deposit a variety of starch granules with changes in morphology and amylopectin structure [274]. Interestingly, FLO6 physically interacted with ISA1, which by itself displayed no binding to starch. It was speculated that FLO6 confers starch binding to ISA1 and that the *flo6* phenotype results from altered ISA1 function. This is an important hypothesis to test given the importance attributed to ISA in trimming nascent amylopectin to facilitate its crystallization.

Another CBM48-containing plastidial protein, Protein Targeting To Starch (PTST), has been described in Arabidopsis [95, 104]. In addition to its CBM, PTST contains predicted coiled-coil motifs. It was shown that PTST binds to GBSS through interaction via a coiled-coil motif on GBSS and that this interaction enabled GBSS to bind to starch granules: loss of PTST resulted in a loss of GBSS from the granule in vivo and loss of amylose synthesis [95]. In addition to the biotechnological relevance (both high and low amylose contents are coveted starch traits), FLO6 and PTST thus constitute exciting examples of non-enzymatic proteins that seem to play an important role in targeting or modulating the activities of starch-biosynthetic enzymes.

Another class of non-enzymatic proteins was very recently discovered in Arabidopsis. Designated Early Starvation1 (EST1), this plastidial starch-binding protein was identified because mutants lacking it display a premature depletion of transitory starch at night [275]. This appears to stem from an alteration in starch granule morphology, resulting in the inappropriate daytime starch degradation and accelerated, uncontrolled starch degradation at night. In contrast, plants overexpressing EST1 developed a starch-excess phenotype indicative of inhibited starch degradation. Although the protein’s precise molecular function is not known, genetic evidence argues against a direct inhibition of degrading enzymes by EST1. It is proposed that EST1 may somehow help glucan molecules align correctly within the granular matrix—a process that has, until now, been regarded to be spontaneous. Interestingly, a homolog of EST1, Like EST1 (LEST), seems to fulfill an opposite role: while *lest* mutants had a wild-type phenotype, overexpression lead to a low-starch phenotype, similar to that of *est1* mutants [275].

Changes in starch granule morphology in cereal endosperms have also been linked to perturbations of other cellular processes including ER stress [276], failure in storage protein trafficking [277] and altered lipid composition of plastid membranes [166]. These observations highlight the dependency of starch granule formation on the overall efficiency of plastid and cellular functioning. Plastid-localized Substandard starch grain4 (SSG4) may also fall into this category. Mutants lacking this protein have enlarged starch grains and plastids, potentially arising from a dysfunction during plastid development [278, 279]. Recently, loss of another stromal protein, Floury Endosperm7 (FLO7), was reported to cause structural and morphological alterations in starch. This phenotype was confined to the periphery of rice seeds where also FLO7 is predominantly expressed, but the function of FLO7 remains unknown [280].

Finally, other studies have begun to uncover regulatory factors that appear to influence starch structure and/or content by controlling the expression of starch-biosynthetic genes. For example, the transcription factors *Os*bZIP58 and Rice Starch Regulator1 (RSR1) were shown to regulate the expression of a series of starch-biosynthetic genes in rice grains, thereby influencing starch structure and granule packing [281, 282]. Similarly, Floury Endosperm2 (FLO2), a nuclear protein with a TPR motif, influences starch granule morphology and the expression of starch-biosynthetic genes, presumably by interacting with transcription factors [283].

This diverse set of newly identified proteins serves to illustrate that there is much still to understand about starch biosynthesis and the cellular context in which it occurs. It is worth noting that most of the proteins described above are evolutionarily conserved within higher plants, and cross-species analyses will be an essential aspect of further investigations.

## Conclusions

Here, we have presented recent research findings in the context of established knowledge and ideas about starch synthesis. Ongoing genetic studies both in model and non-model species continue to raise evidence for enzymatic interdependencies, but also take us closer to understanding the requirements for the synthesis of a crystallization-competent glucan. This is well illustrated by studies of the ISA DBE: its contribution to promoting crystalline starch is highly dependent on the prior action of synthetic enzymes to produce suitable substrates for it, and some aberrant substrates are prone to degradation by ISA, while others are capable of crystallization in its absence. The assembly of enzymes into complexes, emerging as a common theme in cereal storage starch biosynthesis, and the participation of non-enzymatic protein factors add potential new layers of complexity to this already intricate process.

Arguably, one major stumbling block is the lack of a simple heterologous system for starch granule synthesis. We are working towards the production of such a system in the yeast *Saccharomyces cerevisiae* with the vision that this could allow us and others to disentangle starch biosynthesis from concomitant degradation and characterize in detail the functional interplay between starch-biosynthetic enzymes. The demonstration that starch can be made in a heterologous system would also show that we know which enzymatic set(s) are sufficient for starch biosynthesis and stimulate other complementary research. Together with empirical data from in vitro studies, it would represent an excellent starting point for the creation of mathematical models capable of simulating the starch-biosynthetic process. Furthermore, a heterologous system could serve as a valuable tool to aid the targeted modification of our starch crops, given that we are still not able to produce the variety of starches required by industry in planta.

## Abbreviations

AA: Amino acid
ADPglucose: Adenosine 5′-diphosphate-glucose
AGPase: ADPglucose pyrophosphorylase
BE: Branching enzyme
CBM: Carbohydrate-binding module
CLD: Chain-length distribution
DBE: Debranching enzyme
DP: Degree of polymerization
Glc-1-P: Glucose-1-phosphate
Glc-6-P: Glucose-6-phosphate
GBSS: Granule-bound starch synthase
GT: Glycosyltransferase
P_i_: Orthophosphate
PP_i_: Pyrophosphate
SS: Starch synthase
